# Theoretical and Experimental Analysis of Dynamic Characteristics for a Valve Train System

**DOI:** 10.3390/s21196328

**Published:** 2021-09-22

**Authors:** Bo Hu, Yunzhe Li, Lairong Yin

**Affiliations:** 1 Hunan Provincial Key Laboratory of Intelligent Manufacturing Technology for High-Performance Mechanical Equipment, Changsha University of Science and Technology, Changsha 410114, China; hubo4956@csust.edu.cn; 2 State Key Laboratory of Advanced Design and Manufacturing for Vehicle Body, Hunan University, Changsha 410082, China; liyunzhe@hnu.edu.cn

**Keywords:** flexible dynamic model, valve train, cam mechanism, multidirectional deformations, jump and bounce

## Abstract

The valve train is one of the main sources of engine vibration, and its dynamic performance is crucial for output power and fuel consumption. The flexibilities of slender bars and beams should be emphasised in the design of valve trains to develop high-power and high-speed engines with industrial applications. A flexible dynamic model of a valve train system is proposed. In the proposed model, the components, except the cam and gear bodies, are modelled as flexible bodies with multidirectional deformations. The gyroscopic effects of the camshaft, cams and gear discs are also considered to predict dynamic responses at high speeds accurately. Gear meshing, the friction of the cam–tappet pair, the centrifugal force of the cams and valve clearance are also considered. Experiments on housing vibration and pushrod stress are conducted to validate the proposed model. Results show that the proposed model can predict the dynamic stress of the flexible components well and predict the trend shown by the housing vibration. The proposed model shows that excessive cam rotation speed and valve clearance will cause intense bounce and jump phenomena. The proposed model can be an important reference for designing engine work speed, adjusting valve clearance and improving component durability.

## 1. Introduction

The valve train system is one of the most important engine parts. It consists of a series of components that are prone to deformation. The dynamics and kinematics performances of these components affect engine performance indices, including output power, economy, reliability, noise and vibration [1,2], especially at high cam rotation speeds. Therefore, developing a dynamic model that analyses the kinematics and dynamics performances of the valve strain system accurately is important.

Previous studies have developed different dynamic models of the valve train system. Teodorescu et al. [3,4] proposed a dynamic valve train model with two equivalent masses by using the lumped parameter method. Nevertheless, these studies disregard contact deformation between components and the elastic deformation of slender components. A rigid multibody dynamic model of the valve trains or cam mechanisms that considers each component as a rigid [5,6,7,8,9], and many researchers have used this model to optimize the cam profile [10,11]. A multiple degree-of-freedom (DOF) model has been established by dividing the valve spring into multimass elements [12,13]. The distributed parameter model of the valve spring has better accuracy than the lumped parameter model but continues to neglect the elastic deformation of slender components. Jelenschi et al. [7] proposed an improved model that accounts for the flexibility of components. Guo et al. [14] and Qin et al. [15] developed the rigid-flexible coupled dynamic model of the valve train system.

Numerous researchers have applied the flexible dynamics theory to analyse the dynamic performance of mechanical systems. An increasing number of factors are considered in the flexible dynamic model. These factors include the clearance of the follower guide [16] and the friction of the cam–tappet pair [17]. Nevertheless, previous studies have ignored the effect of camshaft dynamics. Rivola et al. [18] developed a dynamic camshaft model by applying the finite element method. The flexibility of the camshaft drastically affects the dynamic behaviour of the valve train [19,20,21]. The gyroscopic effect of the rotor disc [22], the presence of eccentricity [23] and the multidirection vibrations of the camshaft [17,24] have also been considered in the dynamic models of valve trains.

Jump and bounce phenomena [7,12,14,15,24] may occur in the valve train system with the increase in cam speed. Impact forces in the presence of these phenomena cause non-negligible deformation and weaken the dynamic performance of the valve train system. The gyroscopic effect and clearance impact have considerable influence on the dynamic response of the valve train at high speeds. However, previous studies have ignored these two factors. Therefore, a flexible dynamic model with the gyroscopic effect and valve clearance impact was developed in this work. The camshaft and rocker arm were modelled as flexible bodies that are based on Timoshenko beam elements, whereas the slender pushrod, valve and tappet were modelled by using bar elements. In addition, the gear meshing, the friction between the cam and tappet and the centrifugal force of the cams were considered. Then, experiments on the housing vibration and pushrod stress were conducted to validate the proposed model and the effects of the cam speed and valve clearance on jump and bounce phenomena were investigated.

## 2. Dynamic Model

**Figure 1:**
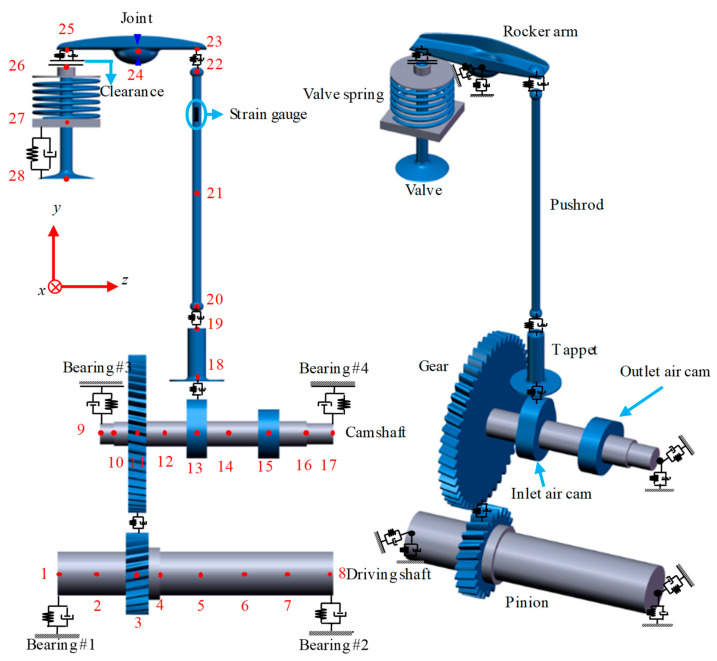
Flexible dynamics model of the valve train system.

A valve train system includes inlet and outlet air valve trains and a camshaft. Given that the structure of the outlet air valve train is the same as that of the inlet air valve train, the outlet air valve train is removed to simplify the dynamic model in this study. The flexible model of the valve train system is presented in Figure 1. The tappet, pushrod and valve are considered as bar elements, whereas the camshaft and rocker arm are divided into the resultant elements of a Timoshenko beam element and bar element. In this study, the gear disc, inlet and outlet air cams, which are mounted on the camshaft, are considered as rigid bodies. The valve spring and the contact deformation between two components are simplified as linear spring-damper elements.

The driving shaft and camshaft are divided into seven and eight elements, respectively, and the inlet air valve train is separated into seven elements. Consequently, the dynamic model includes 28 nodes and 22 elements. The structural parameters of these elements are listed in Table 1.

### 2.1. Flexible Shaft Element

In this work, Timoshenko beam theory is used to establish the flexible shaft element. Given that the camshaft is driven by a helical gear pair, the torsional and axial deformations are included. Therefore, dynamic equations of the resultant element are derived by combining a Timoshenko beam with torsional and bar elements. Each element has 2 nodes, and each node has 6 DOF. The displacement vector of the shaft element is expressed as

(1) $$q_{i}^{s}={\left[\begin{array}{cc} \begin{array}{cc} \begin{array}{cc} \begin{array}{cc} \begin{array}{cc} \begin{array}{ccc} x_{i} & y_{i} & z_{i} \end{array} & \theta_{xi} \end{array} & \theta_{yi} \end{array} & \theta_{zi} \end{array} & x_{\left(i+1\right)} \end{array} & \begin{array}{cc} \begin{array}{ccc} y_{\left(i+1\right)} & z_{\left(i+1\right)} & \theta_{x(i+1)} \end{array} & \begin{array}{cc} \theta_{y(i+1)} & \theta_{z(i+1)} \end{array} \end{array} \end{array}\right]}^{T}$$

where *i* is the node *i* (*i* = 1, 2,⋯, 17) on the camshaft, and the kinetic energy and potential energy of the shaft element are expressed as

(2) $$T^{s}=\frac{1}{2}\int_{0}^{l}\rho A({\dot{x}}_{s}^{2}+{\dot{y}}_{s}^{2}+{\dot{z}}_{s}^{2})ds+\frac{1}{2}\int_{0}^{l}\rho J_{Ds}({\dot{\theta }}_{xs}^{2}+{\dot{\theta }}_{ys}^{2})ds+\frac{1}{2}\int_{0}^{l}\rho J_{Ps}[{\left(\Omega +{\dot{\theta }}_{zs}\right)}^{2}+(\Omega +{\dot{\theta }}_{zs})(\theta_{ys}{\dot{\theta }}_{xs}-\theta_{xs}{\dot{\theta }}_{ys})]ds$$

(3) $$U^{s}=\frac{1}{2}\int_{0}^{l}EJ_{Ds}({\theta^{\prime }}_{xs}^{2}+{\theta^{\prime }}_{ys}^{2})ds+\frac{1}{2}\int_{0}^{l}GJ_{Ps}{\theta^{\prime }}_{zs}^{2}ds+\frac{1}{2}\int_{0}^{l}\mu GA[{\left(x^{\prime }-\theta_{ys}\right)}^{2}+{\left(y^{\prime }+\theta_{xs}\right)}^{2}]ds$$

where the displacement vector of an arbitrary point on the shaft element can be represented as $q_{s}={\left[\begin{array}{cccccc} x_{s} & y_{s} & z_{s} & \theta_{xs} & \theta_{ys} & \theta_{zs} \end{array}\right]}^{T}=Nq_{i}^{s}$, and the shape function **N** can be found in a previous article [25,26]. Besides, ${\dot{\theta }}_{cs}$ (*c* = *x*, *y*, *z*) represents the derivative of $\theta_{\mathrm{cs}}$ with respect to time, however, ${\theta^{\prime }}_{cs}^{}$ (*c* = *x*, *y*, *z*) represents the derivative of $\theta_{\mathrm{cs}}$ with respect to *z* coordinate.

The dynamic equations of the flexible shaft element are derived by using the Lagrange equation, and the matrix form is represented as

(4) $$M_{i}^{s}{\ddot{q}}_{i}^{s}+\Omega G_{i}^{s}{\dot{q}}_{i}^{s}+K_{i}^{s}q_{i}^{s}=F_{i}^{s}$$

The derivation of the matrices **M***_i_^s^*, **G***_i_^s^* and **K***_i_^s^* can be also found in [2].

### 2.2. Rotor Disc and Supporting Stiffness

The rotor discs on the camshaft include the inlet and outlet air cams and the gear body. Six DOF are taken into account and can be represented as

(5) $$q_{i}^{d}={\left[\begin{array}{cccccc} x_{i} & y_{i} & z_{i} & \theta_{xi} & \theta_{yi} & \theta_{zi} \end{array}\right]}^{T}$$

where *i* (*i* = 11, 13, 15) represents the node on which the rotor discs are mounted. The kinetic energy of these discs is represented as

(6) $$T^{d}=\frac{1}{2}[m_{d}({\dot{x}}_{i}^{2}+{\dot{y}}_{i}^{2}+{\dot{z}}_{i}^{2})+J_{Dd}({\dot{\theta }}_{xi}^{2}+{\dot{\theta }}_{yi}^{2})+J_{Pd}{\left(\Omega +{\dot{\theta }}_{zi}\right)}^{2}+J_{Pd}(\Omega +{\dot{\theta }}_{zi})(\theta_{yi}{\dot{\theta }}_{xi}-\theta_{xi}{\dot{\theta }}_{yi})]$$

The dynamics equations of the rotor disc are derived by using the Lagrange equation and can be written as

(7) $$M_{i}^{d}{\ddot{q}}_{i}^{d}+\Omega G_{i}^{d}{\dot{q}}_{i}^{d}=F_{i}^{d}$$

**M***_i_^d^* and **G***_i_^d^* are the mass and gyroscopic matrices, respectively, and are expressed as follows:

(8) $$M_{i}^{d}=\mathrm{diag}\left[\begin{array}{cccccc} m_{d} & m_{d} & m_{d} & J_{Dd} & J_{Dd} & J_{Pd} \end{array}\right]$$

(9) $$G_{i}^{d}=\left[\begin{array}{cccccc} 0 & 0 & 0 & 0 & 0 & 0\\ 0 & 0 & 0 & 0 & 0 & 0\\ 0 & 0 & 0 & 0 & 0 & 0\\ 0 & 0 & 0 & 0 & J_{Pd} & 0\\ 0 & 0 & 0 & -J_{Pd} & 0 & 0\\ 0 & 0 & 0 & 0 & 0 & 0 \end{array}\right]$$

The valve train system has four ball bearings and a joint after removing the outlet air valve train. Bearings #1, #2, #3 and #4 support the camshaft and are installed on nodes 1, 8, 9 and 17, respectively. Joint supports the rocker arm and is mounted on node 24. The camshaft and rocker arm rotate around the *z*-axis. Supporting stiffness **K***^b^* and damping **C***^b^* are represented as [27]

(10) $$K^{b}=\left[\begin{array}{cccccc} k_{xx} & k_{xy} & k_{xz} & k_{x\theta_{x}} & k_{x\theta_{y}} & 0\\ k_{yx} & k_{xx} & k_{yz} & k_{y\theta_{x}} & k_{y\theta_{y}} & 0\\ k_{zx} & k_{zy} & k_{zz} & k_{z\theta_{x}} & k_{z\theta_{y}} & 0\\ k_{\theta_{x}x} & k_{\theta_{x}y} & k_{\theta_{x}z} & k_{\theta_{x}}{}_{\theta_{x}} & k_{\theta_{x}}{}_{\theta_{y}} & 0\\ k_{\theta_{y}x} & k_{\theta_{y}y} & k_{\theta_{y}z} & k_{\theta_{y}}{}_{\theta_{x}} & k_{\theta_{y}}{}_{\theta_{y}} & 0\\ 0 & 0 & 0 & 0 & 0 & 0 \end{array}\right],\;\;\;C^{b}=\left[\begin{array}{cccccc} c_{xx} & c_{xy} & c_{xz} & c_{x\theta_{x}} & c_{x\theta_{y}} & 0\\ c_{yx} & c_{xx} & c_{yz} & c_{y\theta_{x}} & c_{y\theta_{y}} & 0\\ c_{zx} & c_{zy} & c_{zz} & c_{z\theta_{x}} & c_{z\theta_{y}} & 0\\ c_{\theta_{x}x} & c_{\theta_{x}y} & c_{\theta_{x}z} & c_{\theta_{x}}{}_{\theta_{x}} & c_{\theta_{x}}{}_{\theta_{y}} & 0\\ c_{\theta_{y}x} & c_{\theta_{y}y} & c_{\theta_{y}z} & c_{\theta_{y}}{}_{\theta_{x}} & c_{\theta_{y}}{}_{\theta_{y}} & 0\\ 0 & 0 & 0 & 0 & 0 & 0 \end{array}\right]$$

where radial stiffness, tilting stiffness, axial stiffness, radial damping, tilting damping and axial damping are represented as *k_ii_*(*i* = *x*, *y*), *k_θiθi_*, *k_zz_*, *c_ii_*, *c_θiθi_* and *c_zz_*, respectively. The values of these parameters are shown in Table 2. Note that the values of the stiffness (*k_zi_*, *k_iz,_ k_zθi_*, *k_xy_* and *k_yx_*) are small in ball bearings. Thus, the values of these parameters are set as 0.

### 2.3. Gear Meshing Formulations

The camshaft is driven through a helical gear pair. The driving pinion is mounted on node 3, and the driven gear is fixed on node 11. The displacement vector of the helical gear pair is presented as

(11) $$q^{g}={\left[\begin{array}{cc} \begin{array}{cc} \begin{array}{cc} \begin{array}{cc} \begin{array}{cc} \begin{array}{ccc} x_{p} & y_{p} & z_{p} \end{array} & \theta_{px} \end{array} & \theta_{py} \end{array} & \theta_{pz} \end{array} & x_{g} \end{array} & \begin{array}{cc} \begin{array}{ccc} y_{g} & z_{g} & \theta_{gx} \end{array} & \begin{array}{cc} \theta_{gy} & \theta_{gz} \end{array} \end{array} \end{array}\right]}^{T}$$

Taking static transmission error into account, the contact deformation between the meshing gears can be represented as

(12) $$\delta_{m}=V_{g}q^{g}-e_{\mathrm{ste}}(t)$$

where *e*_ste_(*t*) can be defined in accordance with a previous work [28], and vector **V***_g_* can be written as

(13) $$V_{g}=\left[\begin{array}{cc} \begin{array}{cc} \begin{array}{cc} \begin{array}{cc} \begin{array}{cc} \begin{array}{ccc} s_{a}c_{b} & c_{a}c_{b} & s_{b} \end{array} & r_{pb}s_{a}s_{b} \end{array} & r_{pb}c_{a}s_{b} \end{array} & r_{pb}c_{b} \end{array} & -s_{a}c_{b} \end{array} & \begin{array}{cc} \begin{array}{ccc} -c_{a}c_{b} & s_{b} & r_{gb}s_{a}s_{b} \end{array} & \begin{array}{cc} r_{gb}c_{a}s_{b} & r_{gb}c_{b} \end{array} \end{array} \end{array}\right]$$

(14) $$\begin{array}{l} s_{a}=\sin a_{t};\;\;\;c_{a}=\cos a_{t}\\ s_{b}=\sin\beta_{b};\;\;\;c_{b}=\cos\beta_{b} \end{array}$$

The mesh force of the gear pair with backlash can be represented as

(15) $$F_{m}=k_{m}(\gamma_{g0}\delta_{g}+\gamma_{g1}B)+c_{m}\gamma_{g0}{\dot{\delta }}_{m}$$

where the backlash function can be represented as follows:

(16) $$\gamma_{g1}=\{\begin{array}{cc} -1 & \delta_{g}>B\\ 0 & \mathrm{else}\\ 1 & \delta_{g}<-B \end{array}$$

(17) $$\gamma_{g0}=\{\begin{array}{cc} 1 & \left|\delta_{g}\right|>B\\ 0 & \mathrm{else} \end{array}$$

Then, the dynamic equation of the meshing gear pair is expressed in the following matrix form:

(18) $$M^{g}{\ddot{q}}^{g}+C^{g}\gamma_{g0}{\dot{q}}^{g}+K^{g}\gamma_{g0}q^{g}=-k_{m}\gamma_{g1}B{\left(V^{g}\right)}^{T}$$

where the mass matrix **M***^g^*, damping matrix **C***^g^* and stiffness matrix **K***^g^* are written as

(19) $$M^{g}=\mathrm{diag}\left[\begin{array}{cccccccccccc} m_{p} & m_{p} & m_{p} & J_{Dp} & J_{Dp} & J_{Pp} & m_{g} & m_{g} & m_{g} & J_{Dg} & J_{Dg} & J_{Pg} \end{array}\right]$$

(20) $$K^{g}=k_{m}{\left(V_{g}\right)}^{T}V_{g};\;\;\;\;\;\;\;\;\;C^{g}=c_{m}{\left(V_{g}\right)}^{T}V_{g}$$

The time-varying meshing stiffness *k*_m_ using an accumulated integral potential energy method by wan et al. [29]. *c_m_* is calculated by $c_{m}=2\zeta \sqrt{{\bar{k}}_{m}/M_{e}}$. ${\bar{k}}_{m}$ is the average value of *k*_m_ and *M*_e_ can be calculated by $\frac{1}{M_{e}}=\frac{R_{p}^{2}}{J_{Pp}}+\frac{R_{g}^{2}}{J_{Pg}}$. Other parameters of the gear model are listed in Table 3.

### 2.4. Inlet Air Valve Train

The inlet air valve train is divided into seven elements. The tappet, pushrod and valve are meshed as bar elements, and the rocker arm is constructed as a 2D Timoshenko beam element. The dynamic equations of the bar element and 2D beam element can be obtained by extracting the corresponding DOF of the vector $q_{i}^{s}$. The bar element with 2 DOF can be represented as:

(21) $$q_{i}^{b}={\left[\begin{array}{cc} y_{i} & y_{\left(i+1\right)} \end{array}\right]}^{T}$$

where *i* (*i* = 18, 19, 22, 26, 27, 28) is the node number. Six DOF on the rocker arm element are considered and are represented as

(22) $$q_{i}^{k}={\left[\begin{array}{llllll} y_{i} & z_{i} & \theta_{xi} & y_{\left(i+1\right)} & z_{\left(i+1\right)} & \theta_{x\left(i+1\right)} \end{array}\right]}^{T}$$

where *i* (*i* = 23, 24, 25) is the node number on the rocker arm element.

### 2.5. Contact Formula of the Cam–Tappet Pair with Eccentricity

**Figure 2:**
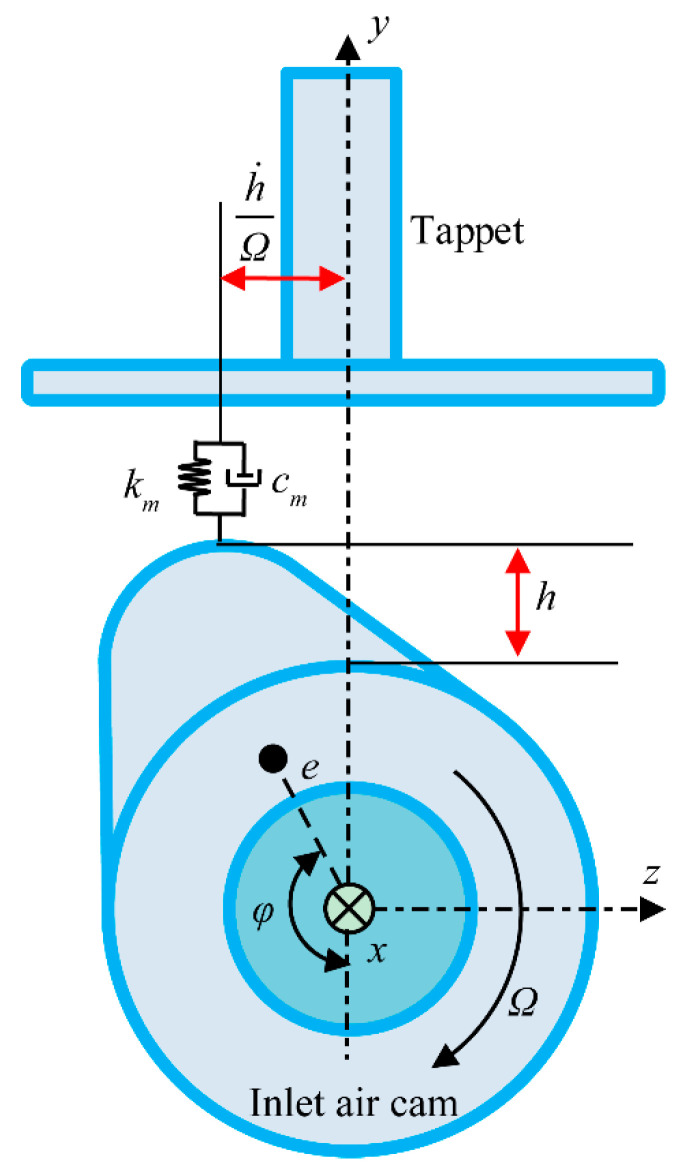
Schematic of the cam–tappet pair.

The camshaft system drives the inlet air train system through the cam-tappet pair. The friction and the centrifugal force of the cam-tappet pair are taken into account. Accoring to Figure 2, *e* is the eccentric distance, and *φ* is the phase angle of the cam. The dynamic contact force *F_c_* and deformation *δ_c_* can be expressed as:

(23) $$F_{c}=\gamma_{c}(k_{c}\delta_{c}+c_{c}{\dot{\delta }}_{c})$$

(24) $$\delta_{c}=Vq^{c}+h(\Omega t)$$

where *k*_c_ and *c*_c_ are obtained in our previous work [30]. ***γ****_c_* is the function that determines whether the cam and tappet are in contact. **q***^c^* is the vector that includes 6 DOFs of node 13 and the axial displacement of node 18. **V** is the vector that determines which DOF in **q***^c^* participates in the calculation of *δ_c_*. ***γ****_c_*, **q***^c^* and **V** are written as

(25) $$\gamma_{c}=\{\begin{array}{c} \begin{array}{cc} 0 & \delta_{c}\leq 0 \end{array}\\ \begin{array}{cc} 1 & \delta_{c}>0 \end{array} \end{array},$$

(26) $$V=\left[\begin{array}{ccccccc} 0 & 1 & 0 & 0 & 0 & 0 & -1 \end{array}\right]$$

(27) $$q^{c}={\left[\begin{array}{ccccccc} u_{73} & u_{74} & u_{75} & u_{76} & u_{77} & u_{78} & u_{103} \end{array}\right]}^{T}$$

The matrix form of the dynamics equations of the cam-tappet pair is expressed as

(28) $$M^{c}{\ddot{q}}^{c}+C^{c}{\dot{q}}^{c}+K^{c}q^{c}=F^{c}$$

In Equation (19), mass matrix **M***^c^*, damping matrix **C***^c^*, stiffness matrix **K***^c^* and force matrix **F***^c^* are written as

(29) $$M^{c}=\mathrm{diag}\left[\begin{array}{ccccccc} m_{c} & m_{c} & m_{c} & J_{dc} & J_{dc} & J_{pc} & m_{16} \end{array}\right]$$

(30) $$C^{c}=\gamma_{c}c_{c}V_{f}^{T}V$$

(31) $$K^{c}=\gamma_{c}k_{c}V_{f}^{T}V$$

(32) $$F^{c}={\left[\begin{array}{cc} \begin{array}{cc} \begin{array}{cc} \begin{array}{cc} \begin{array}{cc} \begin{array}{cc} f_{c}\sin\varphi & -f_{c}\cos\varphi \end{array} & 0 \end{array} & 0 \end{array} & 0 \end{array} & 0 \end{array} & 0 \end{array}\right]}^{T}-\gamma_{c}(k_{c}h+c_{c}\dot{h})V_{f}^{T}$$

where **V***_f_*, *f_c_* and *φ* are written as

(33) $$V_{f}=\left[\begin{array}{cc} \begin{array}{cc} \begin{array}{cc} \begin{array}{cc} \begin{array}{cc} \begin{array}{cc} -\mu & 1 \end{array} & 0 \end{array} & 0 \end{array} & 0 \end{array} & \frac{\dot{h}}{\Omega }+\mu (r_{b}+h) \end{array} & -1 \end{array}\right]$$

(34) $$f^{c}=m_{c}\Omega^{2}e$$

(35) $${\varphi =\Omega t+\varphi }^{0}$$

In Equation (34), the friction coefficient *μ* is predicted on the basis of elastohydrodynamic lubrication theory. The initial phase angle is set as 0 in this work.

### 2.6. Dynamic Equations of the Valve Train System

The overall system equations of the valve train system can be obtained by combining the mass, damping, gyroscopic and stiffness matrices of every node, and the matrix form of overall system equations is expressed as:

(36) $$M\ddot{q}+(C+\Omega G)\dot{q}+\mathrm{Kq}=F$$

where **M**, **C**, **G** and **K** are the overall mass, damping, gyroscopic and stiffness matrices, respectively. **F** is the overall force matrix, and **q** is the vector of the overall DOF.

(37) $$q={\left[\begin{array}{cccccc} u_{1} & u_{2} & u_{3} & \ldots & u_{121} & u_{119} \end{array}\right]}^{T}$$

## 3. Experimental Verification and Discussion

**Figure 3:**
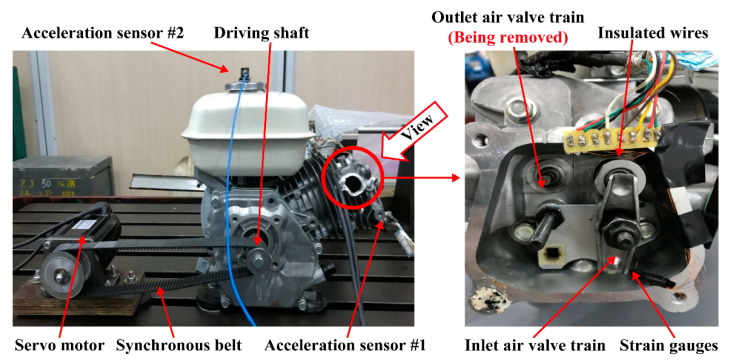
Arrangement of the test rig for measuring housing vibration and pushrod stress.

Housing vibration and pushrod stress were measured by using a test rig of the valve train system. In Figure 3, the camshaft of the valve train system was driven by the servo motor with synchronous belt transmission, and the rotation speed of the camshaft was controlled by the motor. The piston, crank shaft and connecting rod affect housing vibration. Thus, these components were removed from the test rig. The outlet train was also removed from the test rig given that this work only analyses the dynamic behaviours of the inlet air valve train and camshaft.

Sensor #1 and Sensor #2 are two triaxial acceleration sensors. Their positions are illustrated in Figure 3. Sensor #1 was utilised to measure the housing acceleration near the joint. Sensor #2 was also used to measure the three-directional acceleration of the housing. However, the three directions did not correspond with the three directions of the global coordinate system (Figure 1), and some processing errors were generated during coordinate transformation processing. Therefore, the test data from sensor #2 were not used in this study. The sensitivity of acceleration sensor is 96.3 mV/g, and the measuring range is from −10 g to 10 g, where g is the acceleration of gravity. The internal excitation current source is used in acceleration, and the excitation current is 4 mA. The sampling frequency of acceleration measurement is set to 4096 Hz.

A full-bridge converter comprising four strain gauges mounted on the pushrod was utilised to measure pushrod stress. The installation positions of the four strain gauges have been previously described [31]. The strain gauge factor is 1.76, and the strain gauge resistance is 120 ± 3 Ω. Before installation, the real resistances of all gauges are measured using a digital multi-meter and is inputted in the LabVIEW system. The excitation voltage is set to 2.5 V and the sampling frequency of strain measurement is 10 kHz. Note that the initial strain of the four strain gauges must be balanced before measurement.

### 3.1. Housing Vibration

**Figure 4:**
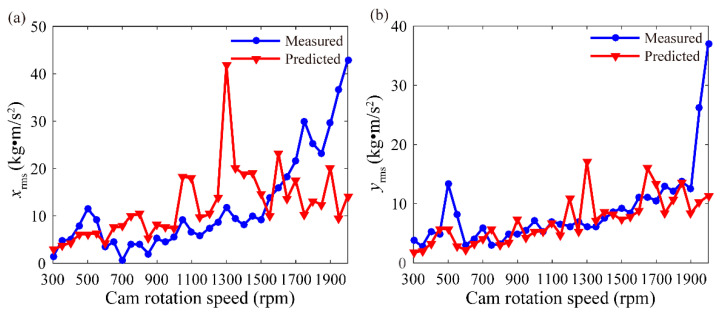
Comparison of the measured and predicted vibration at cam speed of 300–2000 rpm: (**a**) direction x and (**b**) direction y.

The vibration of the valve train system is transmitted to the housing through the bearings and joint. Housing vibration was evaluated by using the root-mean-square (RMS) of acceleration during many periods. The test data of Sensor #1 were obtained at the cam rotation speed of 300–2000 rpm to verify the predicted acceleration on node 24. The results for the comparison between the RMS values of the predicted and measured accelerations are illustrated in Figure 4, where the moving average method is applied to eliminate the white noise of the measured vibration signals.

As shown in Figure 4a, *x*_rms_ is the RMS of the acceleration along the *x* direction. The trend shown by the predicted vibration is the same as that shown by the measured vibration when the cam rotation speed ranges from 300 rpm to 1450 rpm. In detail, the two curves present the same five vibration peaks at the corresponding speeds of 500, 750, 900, 1050 and 1300 rpm. A sharp upward trend appears in the measured curve, whereas the predicted curve presents a different trend when the speed exceeds 1450 rpm. The predicted vibration near 1300 rpm is higher than the measured vibration. This result may be attributed to the constant empirical damping used in this work given that the damping of the valve train system is difficult to predict accurately.

In Figure 4b, *y*_rms_ represents the RMS of the acceleration along the *y* direction. Overall, the measured curve shows a gradual upward trend. A large, distinct vibration peak is generated at the speed of 500 rpm, and the vibration increases sharply at 1900 rpm to 2000 rpm. The predicted curve also presents a slow upward trend and a vibration peak near the speed of 500 rpm. By contrast, the predicted curve exhibits additional vibration peaks at the speed of 1200 and 1300 rpm. The amplitudes of the two curves show some differences because the predicted vibration represents the RMS of the acceleration on node 24, whereas the measured vibration represents the RMS of the acceleration on the engine housing (Figure 3).

**Figure 5:**
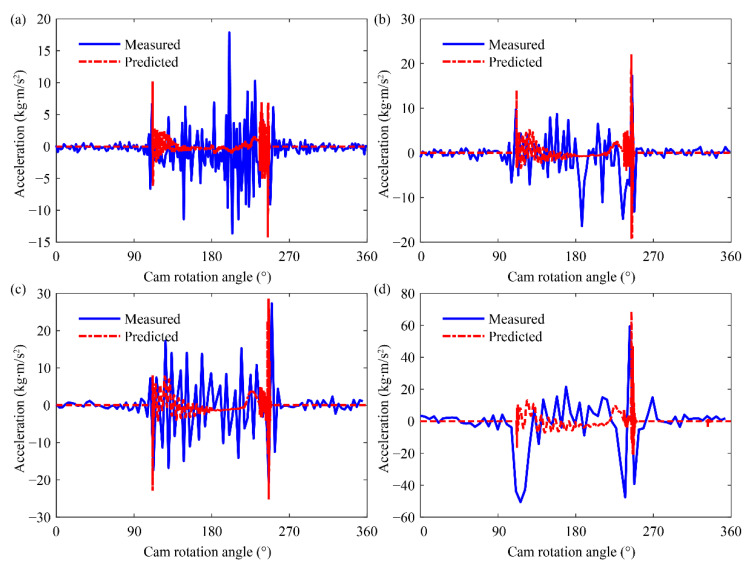
The measured and predicted acceleration at (**a**) 600 rpm, (**b**) 900 rpm, (**c**) 1200 rpm and (**d**) 1800 rpm.

Figure 5 illustrates the measured and predicted accelerations at different rotation speed. The acceleration becomes large with an increase in rotation speeds. Although the bars do not move during the dwell phase, the measured accelerations also have fluctuations. These fluctuations are caused by the synchronous belt and gear transmission. The predicted values are the acceleration on node 24, which does not include the synchronous belt and gear vibration. Therefore, there is no fluctuation in the curves of the predicted acceleration during the dwell phase. When the cam begins to enter or exit the working phase, two peaks appear in the curves of the acceleration. The first peak is caused by the valve impact due to the valve clearance, and the valve impact can even generate jump phenomenon of the valve. The second peak is induced by the repeated pulling and pressing of the valve spring, which causes the bounce phenomenon of the valve. The jump and bounce phenomena will be discussed in detail in the following section.

### 3.2. Pushrod Stress

**Figure 6:**
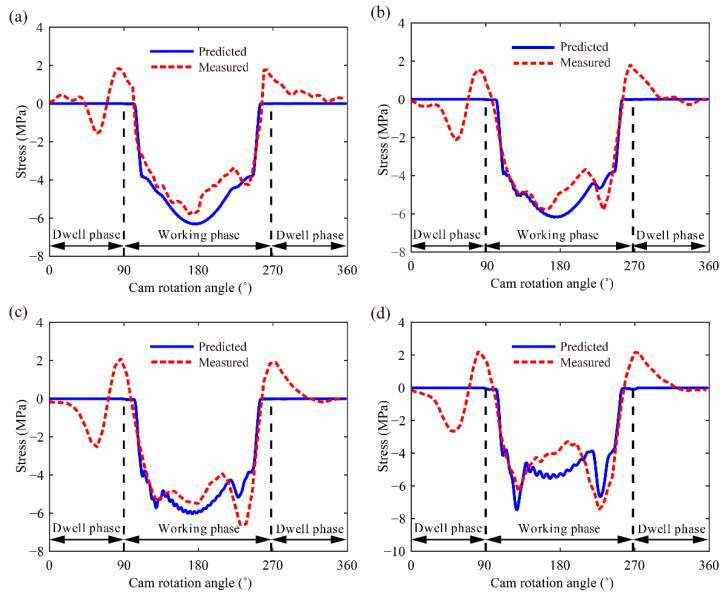
Predicted and measured stresses at (**a**) 600, (**b**) 900, (**c**) 1200 and (**d**) 1800 rpm.

The proposed model can be used to predict the dynamic stress of the components in the valve train system, and pushrod stress can be predicted by $\sigma \;=\;E\left(u_{107}-\;u_{106}\right)/L_{18}$. Figure 6 shows the comparison results of the predicted and measured pushrod stresses at the cam rotation speeds of 600, 900, 1200 and 1800 rpm.

Positive and negative values indicate that the pushrod is stretched and compressed, respectively. During the working phase, the predicted pushrod stress first increases and then decreases at the speed of 600 rpm (Figure 6a). In contrast to the predicted results, a peak appears at approximately 235° in the measured curve at 600 rpm. As shown in Figure 6b–d, two peaks appear simultaneously in the predicted and measured curves at approximately 130° and 235°. This is because the dynamic effect on contact force and elastic deformation is considered in this work. The cam curvatures at these two positions change significantly and cause two large accelerations, which can be seen in [31]. The acceleration and contact force become large with increase in cam rotation speeds, and the contact force at these two positions will even exceed the contact force at 180°. Therefore, the stresses at these two positions increase with the increment in cam rotation speed and even exceed the stress at 180°. Slight differences exist between the predicted and the measured curve, but the two curves follow similar trends during the working phase.

The value of the predicted stress is approximately 0 during the dwell phase at four different cam rotation speeds. However, although the measured data have been processed by using the time-domain synchronous averaging method, large peaks appear in the measured curves, and the tensile stress also appears during the dwell phase. In fact, the pushrod only bears pressure. The elastic compression of the pushrod releases rapidly and results in a transitory stretch, thereby causing the tensile stress. However, the predicted model does not yet take this into account, which causes the existence of differences between the predicted and measured stresses.

### 3.3. Bounce and Jump

The dynamics and kinematics responses of the valve have drastic effects on the inlet and exhaust of the valve trains. The occurrence of abnormal phenomena, such as valve bounce and jump, with the increase in cam rotation speed will reduce the output power of the engine, shorten the service life of the valve spring seat and increase vibration and noise. Therefore, valve lift curves at different cam rotation speeds are predicted by using the proposed model and are compared with the theoretical lift curve to analyse the valve bounce and jump phenomena wherein the theoretical cam lift is obtained by the geometry.

**Figure 7:**
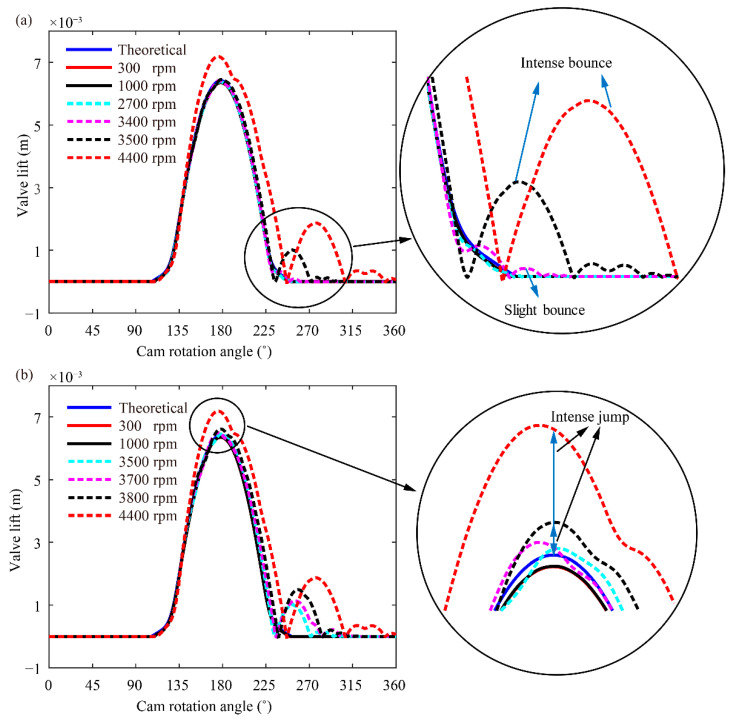
Bounce and jump at different cam rotation speeds: (**a**) bounce and (**b**) jump.

Figure 7 shows the bounce and jump phenomena that occur at different cam rotation speeds when the valve clearance is set as 0.15 mm. Valve bounce is a phenomenon wherein the valve repeatedly impacts the valve seating. Valve jump is a phenomenon wherein the excessive inertial force of the valve results in the separation of the valve and rocker arm. In theory, the valve impact during the rise phase can cause the jump phenomenon, but the inertial of the valve is the dominant factor for this valve train. As shown in Figure 7a, valve bounce and slight bounce do not occur when the cam rotation speed is lower than 3400 rpm. However, intense bounce occurs when the cam rotation speed reaches 3500 rpm. In addition, the jump and bounce are also closely related to the performance parameters of the valve spring.

In general, the dynamic valve lift predicted by the flexible model should be lower than the theoretical dynamic valve lift because of elastic deformation (at 300 and 1000 rpm in Figure 7b). Valve jump occurs when the cam rotation speed is increased to 3500 rpm because of the separation between the valve and rocker arm. Slight jump occurs when the cam rotation speed is in the range of 3500–3700 rpm. Intense jump occurs when the cam rotation speed exceeds 3800 rpm. Bounce and jump phenomena at different cam rotation speeds are listed in Table 4.

As illustrated in Table 4, the cam rotation speed has a drastic effect on valve bounce and jump. Only slight bounce with negligible effects on dynamic behaviour is observed at the speed of 3400 rpm. Slight jump and intense bounce are observed at the speed of 3500 rpm. Jump sharply increases the degree of valve bounce by introducing large impact forces between the valve and seat at seating. Moreover, it delays valve closing. Therefore, jump will increase vibration; reduce output power and damage the valve, spring and seat. The predicted results indicate that the working speed of the valve train system should not exceed 3400 rpm.

The generation of impact force between the rocker arm and valve given the existence of valve clearance aggravates the degree of bounce and jump. The valve lift curves at different clearances are predicted at the cam rotation speed of 3400 rpm to investigate the effect of valve clearance on bounce and jump.

**Figure 8:**
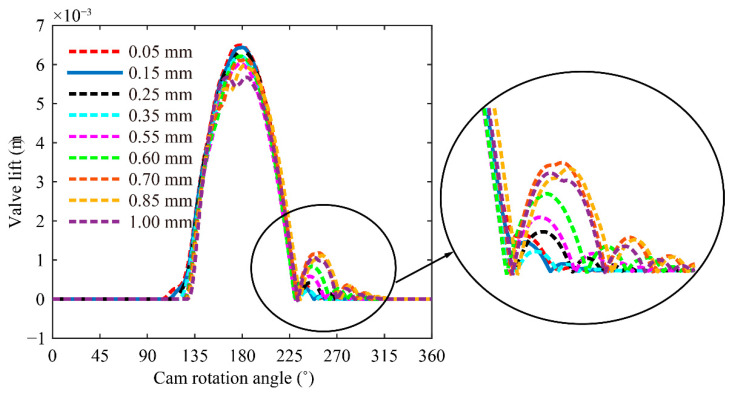
Comparison of the bounce phenomenon at different valve clearances.

Figure 8 illustrates the degree of valve bounce at different clearances. Bouncing degrees show slight differences when valve clearance < 0.55 mm and increase when valve clearance is increased to 0.55 mm. The increase in valve clearance to a certain degree will cause excessive bounce. However, bounce is attenuated when the valve clearance exceeds 0.7 mm. Given that the maximum compression of the valve spring is reduced with the increase in clearance, the effect of reduced spring force on the bounce exceeds that of impact force caused by clearance. This effect weakens bounce.

**Figure 9:**
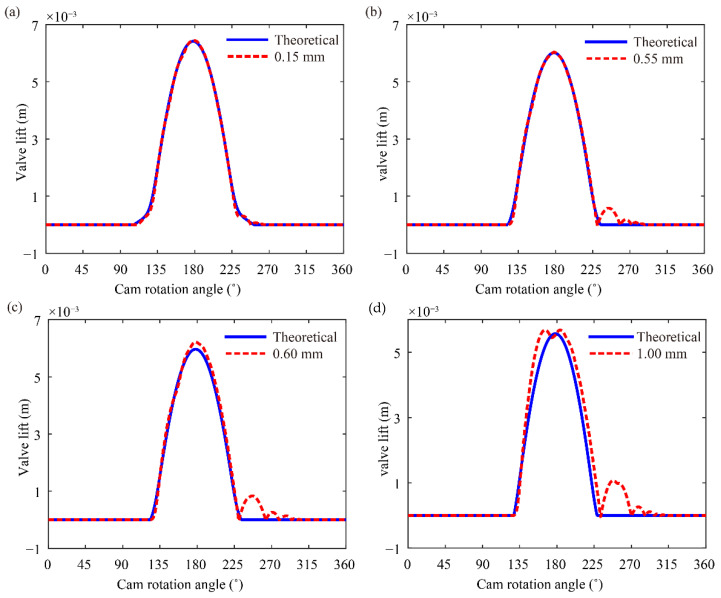
Comparison of the jump phenomenon at different valve clearances: (**a**) 0.15 mm, (**b**) 0.55 mm, (**c**) 0.60 mm and (**d**) 1.00 mm.

The degree of jump at different clearances is shown in Figure 9. Intense jump occurs when valve clearance exceeds 0.55 mm, and the degree of jump is aggravated with the increase in the valve clearance. The contact and separation states between the cam, tappet, pushrod, rocker arm and valve are included in the proposed model. Impact force is closely related to impact velocity, as shown in Equations (23)–(25). The impact velocity increases when crossing a large clearance, and the valve spring is difficult to restrain separation between two parts. The theoretic valve lift is larger than the predicted one with a small clearance due to the elastic deformation. Although there is also the clearance and elastic deformation, the theoretic valve lift is smaller than the predicted one with a large clearance because of the existence of separation. Therefore, the excessive valve clearance will cause intense jump due to the separation.

## 4. Conclusions

A flexible dynamic model with the gyroscopic effect and valve clearance impact was developed in this work. The camshaft and rocker arm were modelled as flexible bodies that were based on Timoshenko beam elements, whereas the slender pushrod, valve and tappet were modelled by using bar elements. This model accounted for the multidirectional deformation of the rocker arm; the torsion-bending coupling vibration of the camshaft; the gyroscopic effect of the camshaft, cam and gear discs; gear meshing; the friction between the cam and tappet and the centrifugal force of the cams. Then, the proposed model was verified through experiments on housing vibration and pushrod stress. Finally, the bounce and jump phenomena were predicted by the proposed model, and the effects of the cam speed and valve clearance on the severity of jump and bounce were investigated.

(1) The comparative analysis of the measured and predicted housing vibrations shows that the proposed model can predict the vibration trend. The differences between the measured and predicted results may be attributed to the difference between the measured position and the predicted node. In future studies, housing will be meshed as a flexible component through the finite element method, and housing vibration at the same measurement position will be predicted.

(2) Given that the components were modelled as flexible bodies, the proposed model can be used to predict inner stress. Some small differences existed between the amplitudes of the measured stress and predicted pushrod stresses. The comparative analysis indicates that the proposed model can predict the dynamic stress of the flexible components well. In addition, the increment in impact force and inertial force at approximately 130°and 230° with the increase in cam speed caused two peaks in the pushrod stress.

(3) The cam rotation speed and valve clearance had a considerable effect on the jump and bounce phenomena of the valve train. Intense jump and bounce phenomena occurred when the cam speed exceeded 3500 rpm. Excessive valve clearance resulted in intense jump and bounce even when the valve train worked at low speed. In addition, jumping caused intense bounce because of the separation of the cam and tappet.

The proposed model can be applied to predict the dynamic performance of valve trains. The analytical results are beneficial for adjusting valve clearance, designing cam profiles, improving component durability and reducing experimental costs. The accuracy of the prediction results can be improved by considering manufacturing and assembly errors in the proposed model. This approach will be studied in future works.

## Figures and Tables

**Table 1:** Structural parameters of the shaft elements.

Element No.	Diameter (mm)	Length (mm)	Element No.	Diameter (mm)	Length (mm)	Element No.	Diameter (mm)	Length (mm)
Driving shaft			Camshaft			Inlet air valve train		
➀	25.00	13.00	➇	12.00	6.20	⑯	8.00	30.50
➁	25.00	13.00	➈	14.00	10.20	⑰	4.00	67.00
➂	30.00	11.00	⑩	14.60	12.85	⑱	4.00	67.00
➃	14.00	18.71	⑪	14.60	13.35	⑲	6.50	26.67
➄	14.00	18.71	⑫	14.60	15.70	⑳	6.50	30.67
➅	14.00	18.71	⑬	14.60	15.90	㉑	5.50	32.00
➆	12.00	18.71	⑭	14.60	16.78	㉒	5.50	32.00
			⑮	12.00	11.26			

**Table 2:** Values of bearing stiffness and damping.

Bearings Stiffness	Bearing #1 and #2	Bearing #3 and #4	Joint
*k_xx_* (N/m)	6.8 × 10^7^	4.1 × 10^7^	1.1 × 10^8^
*k_yy_* (N/m)	6.8 × 10^7^	4.1 × 10^7^	1.1 × 10^8^
*k_zz_* (N/m)	5.4 × 10^6^	1.9 × 10^7^	1.1 × 10^6^
*k_θxθx_* (N/rad)	2.1 × 10^3^	1.9 × 10^3^	/
*k_θyθy_* (N/rad)	2.1 × 10^3^	1.9 × 10^3^	/
*k_xθy_*_,_*k_yθx_*_,_*k_θxy_*_,_*k_θyx_* (N/rad)	3.7 × 10^5^	2.8 × 10^5^	/
*c* (Ns/m)	1.0 × 10^3^	1.0 × 10^3^	1.0 × 10^3^

**Table 3:** Parameters of the helical gear pair.

Parameters	Values	Parameters	Values
*m* (mm)	1.75	*J_Pp_* (kg∙m^2^)	2.13 × 10^−5^
*m_p_* (kg)	6.00 × 10^−2^	*J_Pg_* (kg∙m^2^)	3.51 × 10^−4^
*m_g_* (kg)	3.61 × 10^−1^	*R_p_* (mm)	21.74
*z_p_*	24	*R_g_* (mm)	43.48
*z_g_*	48	*α_n_* (°)	20
*J_Dp_* (kg∙m^2^)	1.12 × 10^−5^	*β* (°)	15
*J_Dg_* (kg∙m^2^)	1.77 × 10^−4^	*ζ*	0.07

**Table 4:** Bounce and jump phenomena at different cam rotation speeds.

Cam Rotation Speed	Bounce Phenomenon	Jump Phenomenon
300 rpm	No bounce	No jump
1000 rpm	No bounce	No jump
2700 rpm	No bounce	No jump
3400 rpm	Slight bounce	No jump
3500 rpm	Intense bounce	Slight jump
3700 rpm	Intense bounce	Slight jump
3800 rpm	Intense bounce	Intense jump
4400 rpm	Intense bounce	Intense jump

## Data Availability

All experimental and predicted data will be made available on request to the correspondent author’s email with appropriate justification.

## Nomenclature

*c_m_*	Mesh damping of the gear pair
*c_c_*	Contact damping of the cam-tappet pair
*e*_ste_(*t*)	Static transmission error
*f_c_*	Cam centrifugal force
*k_m_*	Mesh stiffness of the gear pair
*k_c_*	Contact stiffness of the cam-tappet pair
*m_c_*	The mass of the cam
*m_p_*	The mass of the pinion
*m_g_*	The mass of the gear
*m_d_*	The mass of the disc
*m_i_*	The mass of *i*th element
**q**	Displacement vector of the overall DOF
**q** *_i_^s^*	Displacement vector of the shaft element
**q** *_s_*	Displacement vector of arbitrary point on the shaft
**q** *^g^*	Displacement vector of the meshing gear pair
**q** *_i_^d^*	Displacement vector of node *i* on the rotor disc
**q** *_i_^k^*	Displacement vector of node *i* on the beam element
**q** *_i_^b^*	Displacement vector of node *i* on the bar element
**q** *^c^*	Displacement vector of the cam-tappet pair
*r_bp_*	The base radius of the pinion
*r_bg_*	The base radius of the gear
*u_i_*	The *i*th DOF of the dynamic model
*A*	The cross-sectional area of the shaft element
*B*	The half of gear backlash
**C** *^b^*	Damping matrix of the bearing
**C** *^g^*	Damping matrix of the gear pair
**C** *^c^*	Damping matrix of the cam-tappet pair
**C**	Overall damping matrix of the dynamic model
*E*	Young’s modulus of the material
**F** *_i_^s^*	Force matrix of the shaft element
**F** *_i_^d^*	Force matrix of the rotor disc
*F*_c_	Contact force of the cam-tappet pair
**F** *^c^*	Force matrix of the cam-tappet pair
**F**	Overall force matrix of the dynamic model
**G**	Overall gyroscopic matrix of the dynamic model
**G** *_i_^s^*	Gyroscopic matrix of the shaft element
**G** *_i_^d^*	Gyroscopic matrix of the rotor disc
*J_Ds_*	Diameter mass moments of inertia of the shaft element
*J_Ps_*	Polar mass moments of inertia of the shaft element
*J_Dd_*	The moment of inertia about a diameter of the disc
*J_Pd_*	The polar moment of inertia of the disc
*J_Pp_*	The polar moment of inertia of the pinion
*J_Dp_*	The moment of inertia about a diameter of the pinion
*J_Dg_*	The moment of inertia about a diameter of the gear
*J_Pg_*	The polar moment of inertia of the gear
*J_Pc_*	The polar moment of inertia of the cam
*J_Dc_*	The moment of inertia about a diameter of the cam
**K** *_i_^s^*	The stiffness matric of the shaft element
**K** *^b^*	The stiffness matric of the ball bearing
**K** *^g^*	Stiffness matrix of the gear pair
**K** *^c^*	Stiffness matrix of the cam-tappet pair
**K**	Overall stiffness matrix of the dynamic model
*L_i_*	The length of *i*th element
**M** *_i_^s^*	Mass matrix of the shaft element
**M** *_i_^d^*	Mass matrix of the rotor disc
**M** *^g^*	Mass matrix of the gear pair
**M** *^c^*	Mass matrix of the cam-tappet contact
**M**	Overall mass matrix of the dynamic model
**N**	Shape function of the shaft element
*α_t_*	Transverse pressure angle
*β_b_*	Base helix angle
*δ_m_*	Dynamic displacement of the meshing gear pair
*δ_c_*	Dynamic deformation of the cam-tappet pair
*μ*	Friction coefficient of the cam-tappet pair
*ρ*	Material density of the shaft element
*φ*	Phase angle of the cam
*φ* ^0^	Initial phase angle of the cam
*Ω*	Spin speed of the shaft
